# When Brownian Motion Meets Clinical Laboratory Automation: A DLS-Inspired Autocorrelation Function for Characterizing Workflow Performance in Sample Processing

**DOI:** 10.3390/diagnostics16132120

**Published:** 2026-07-07

**Authors:** Claudia Spoliti, Raimondo De Cristofaro, Enrico Di Stasio

**Affiliations:** 1 Department of Translational Medicine and Surgery, Catholic University of the Sacred Heart, Largo Francesco Vito 1, 00168 Rome, Italy; claudia.spoliti@unicatt.it (C.S.); raimondo.decristofaro@unicatt.it (R.D.C.); 2 Foundation University Hospital “A. Gemelli” IRCCS, Largo Agostino Gemelli 8, 00168 Rome, Italy; 3 Department of Basic Biotechnological Sciences, Intensive Care and Perioperative Clinics, Catholic University of the Sacred Heart, Largo Francesco Vito 1, 00168 Rome, Italy

**Keywords:** dynamic light scattering, correlation function, automation chain, sample flow, turnaround time, laboratory efficiency, laboratory performance, laboratory workflow

## Abstract

**Background/Objectives**: Laboratory automation is a key strategy for increasing productivity and reducing sample turnaround time (TAT), a common indicator of laboratory performance. However, owing to the statistical distribution of TAT values, conventional descriptors such as mean, standard deviation, and percentiles cannot capture the processing history of individual samples. In this study, sample flow within a highly automated laboratory system was analyzed by analogy with the Brownian motion of molecules in solution, using an ad hoc modified Dynamic Light Scattering (DLS) correlation function. **Methods**: Seven processing histories, each consisting of 1000 samples and representing different TAT scenarios, were generated, and the corresponding correlation functions were calculated. Each sample was assumed to remain correlated with its initial state (value = 1) until its TAT was reached; thereafter, once the result was produced, the sample was considered uncorrelated and its status value became 0. The correlation function was defined as the normalized progressive sum, over time, of the status values of all analyzed samples at each time point. **Results**: The DLS-inspired autocorrelation function enabled the derivation of parameters describing both overall system performance and sample processing status. These parameters provide quantitative indicators for near-real-time monitoring of automation chain efficiency and reveal system features that are not accessible through conventional TAT statistics. **Conclusions**: This approach allows the definition of measurable metrics describing the system’s capacity to buffer and mitigate operational disruptions at both the global and individual-sample levels. The proposed framework provides a novel tool for evaluating, monitoring, and comparing the performance of laboratory automation systems.

## 1. Introduction

Laboratory automation represents a major development in modern clinical laboratories [1,2,3,4]. It can be defined as a multidisciplinary approach that integrates robotics, circuitry, informatics, engineering, medicine, and chemistry to optimize laboratory workflows from the collection of biological samples to the generation of clinically relevant results [1,5]. In clinical settings, automation now spans multiple specialized areas, including clinical chemistry, coagulation, hematology, immunology and immunochemistry, urinalysis, transfusion medicine, microbiology, mass spectrometry, and molecular diagnostics [1,4,5]. The primary goal of laboratory automation is to enhance productivity and shorten process cycle times, ultimately improving efficiency, accuracy, and data quality [6,7,8]. Automated platforms consist of robotic systems that combine instruments, autosamplers, and dedicated software into a single operational chain managing the analytical process from sample arrival to result reporting. These systems operate according to a “fire-and-forget” approach: once samples are loaded, the platform automatically identifies and processes them with minimal human intervention, ensuring process standardization while minimizing variability and errors associated with manual handling [1,5,9]. Samples that do not meet processing requirements—due to issues such as electronic request errors, identification problems, or sample clotting—are classified as non-compliant and set aside for later evaluation by laboratory personnel. However, in routine practice, laboratory workflows are often more complex than theoretical models, and additional handling steps may still introduce processing delays.

The sample turnaround time (TAT) is a key performance indicator (KPI) widely used to assess the performance and efficiency of the diagnostic process [10,11]. It comprises three main phases: pre-analytical (from test ordering to sample receipt in the laboratory), analytical (intra-laboratory processing), and post-analytical (result validation, reporting, and communication) [12]. Therefore, TAT is a critical determinant of healthcare quality, linking laboratory testing to clinical decision-making and therapeutic action [13,14]. Among these phases, the analytical component is the most directly controllable and used to assess internal laboratory performance [15]. Analytical TAT includes all intra-laboratory activities from sample check-in (recorded as “received” via barcode scanning) to the release of the final report to clinicians, encompassing steps such as electronic registration, transport tracking, analytical sampling and processing, and result generation [11,16]. Each of these steps has a theoretical minimum duration (i.e., the fastest achievable time). For instance, if a centrifuge is programmed for a 10 min run, the actual duration may still increase due to delays such as balancing issues. Because each phase has a minimum achievable time and delays can only extend the duration, the distributions of the individual steps and of the total TAT are typically non-Gaussian [12]. Consequently, the use of the mean and standard deviation is not appropriate for describing TAT distributions. Instead, TAT data generally show a positively skewed distribution (with a right-hand tail), making the median and the tail-related metrics more suitable descriptive measures [17]. Tail behavior can be characterized in different ways, for example by calculating the proportion of samples exceeding a predefined time threshold (outlier rate) or by determining the time corresponding to a given percentile of the distribution (e.g., the 90th percentile) [17]. The latter measure is increasingly reported in the literature and is commonly referred to as the 90% completion time [15,18,19,20,21]. An alternative strategy involves applying failure-time analysis methods to TAT data, including Kaplan–Meier survival curves, log-rank tests, and Cox proportional hazards models [17,22]. In this framework, samples are treated analogously to patients in survival analysis. At sample registration, the TAT clock is set to zero. When processing is completed, the sample is considered analogous to a “death event”, and the elapsed time from registration corresponds to the sample’s “survival time”. This approach enables comparison of different sample categories (e.g., routine vs. urgent samples) using log-rank tests and allows identification of variables influencing TAT through Cox regression models. However, despite its analytical value, this methodology is generally less practical for routine monitoring of TAT performance [17,23]. Typically, only a small proportion of samples (usually less than 10%) are rejected; however, the main concern is the time required to alert laboratory personnel, particularly when emergency samples are involved. In addition, the difficulty of tracking individual specimens throughout their progression within the analytical system can lead to inadequate monitoring and control of TAT [23]. Consequently, despite the widespread use of TAT as a KPI, current approaches provide limited insight into the dynamic behavior of sample flows within automated laboratory systems.

Brownian motion refers to the random movement of particles dispersed in a liquid medium [24]. This motion can be monitored using techniques such as Dynamic Light Scattering (DLS), which detects temporal fluctuations in the intensity of light scattered by the sample, I(t) [25,26,27]. The rate of these fluctuations around the mean intensity reflects the speed at which the scattering particles diffuse in solution. This motion is described by an apparent diffusion coefficient, D_app_ [25,26]. In a DLS experiment, the primary experimental output is the autocorrelation function of the photodetector current. This autocorrelation curve contains information related to the diffusive motion of the particles. By fitting the correlation function with an exponential decay model, the diffusion coefficient can be determined, since the diffusion coefficient is related to the characteristic decay time of the exponential function. Once the diffusion coefficient has been obtained, the hydrodynamic diameter of the particle can be estimated using the Stokes–Einstein equation [25,26,28]. As previously stated, the autocorrelation function is obtained using a digital correlator, which evaluates the degree of similarity between two signals as a function of time. When the scattered light intensity measured at a given time (t_0_) is compared with the intensity measured a very short interval later (t_0_ + δt), the two signals are highly similar and therefore strongly correlated. If the comparison is made at a slightly longer delay time (t_0_ + 2δt), the signals remain related but the degree of correlation is reduced compared with the earlier interval. Thus, the correlation progressively decreases as the delay time increases. At sufficiently long delay times, the signals become completely unrelated because of the random Brownian motion of the particles, resulting in the disappearance of correlation [25,26]. In DLS measurements, these processes occur on very short time scales. For a typical speckle pattern, the correlation function generally decays to zero within approximately one to several tens of milliseconds. If the intensity signal at time t is compared with itself, the correlation is perfect because the two signals are identical. In this case, the correlation value is defined as 1, whereas a value of 0 corresponds to the absence of correlation [27]. When larger particles are present, their slower diffusion in solution results in slower fluctuations in the speckle intensity pattern. In contrast, smaller particles diffuse more rapidly, leading to faster fluctuations in the scattered light intensity [29].

From a conceptual perspective, despite the obvious differences between microscopic particle motion and macroscopic laboratory workflows, the movement of samples along an automated laboratory track can be viewed as a stochastic process analogous to the random walk of particles in a fluid. Accordingly, principles and analytical approaches commonly used in biotechnology and thermodynamic theory to describe the microscopic behavior of particles are applied here to the macroscopic process of blood sample handling in automated laboratory systems, with the objective of gaining new insights into the management and efficiency of laboratory workflows involved in producing clinically relevant analytical results.

The proposed method is not intended to replace survival analysis, but rather to recast the empirical survival function into a discrete-time operational framework suitable for continuous monitoring of laboratory workflow. Inspired by the autocorrelation function used in DLS analysis, the proposed approach introduces an analytical function that mathematically describes the temporal evolution of sample completion by quantifying the progressive decrease in the fraction of samples remaining under processing, thereby providing a simple graphical and quantitative representation of the dynamic clearance process.

Within this framework, the similarities and differences between the microscopic-scale systems investigated by DLS and the large-scale processing of blood samples in automated laboratory tracks are examined and discussed. Furthermore, experimentally measurable parameters are introduced to evaluate the system’s ability to buffer and compensate for TAT delays caused by problematic samples, providing practical metrics for assessing the overall analytical workflow performance and facilitating straightforward comparison between different operational configurations. In particular, the proposed framework enables the definition of user-defined operational checkpoints, allowing deviations from expected workflow to be quantified and monitored throughout the analytical process.

## 2. Materials and Methods

### 2.1. Definition of TAT and Data Generation

As outlined in Section 1, intra-laboratory TAT is defined as the interval between sample entry into the laboratory automation system and completion of the analytical process, when results become available for clinical reporting [11,16,17]. The proposed approach can be readily extended to different choices of initial and final time points.

Data (TAT values, expressed in minutes) were generated in Microsoft Excel (Microsoft 365, Microsoft Corp., Redmond, WA, USA) using the built-in pseudorandom number generator RAND(), which produces values uniformly distributed between 0 and 1. The generated values were subsequently transformed to match the desired range and distribution according to the specific case under study. For each condition, 1000 TAT values were generated to ensure adequate statistical representation.

### 2.2. Correlation Function and Theoretical Framework

These data were then used to derive the correlation function according to the following Equation (discrete time):

(1) $$G_{exp}\left(t\right)=\frac{1}{n}\sum_{x=1}^{n}\frac{\left|t_{i}^{x}-\tau_{x}\right|-\left(t_{i}^{x}-\tau_{x}\right)}{2\left(\left|t_{i}^{x}-\tau_{x}\right|+\varepsilon \right)}\;t\in \{0,1,2,\ldots ,t_{\max}\}$$

where n is the total number of samples under observation, and t is a discrete variable ranging from 0 to t_max_ with a one-minute step. Accordingly, t_i_^x^ represents the ith time channel (1 min interval) for each sample x. τ_x_ is the TAT of sample x, and ε is a small positive constant introduced to avoid indeterminate equation solutions when t_i_^x^ = τ_x_. In all analyses, ε was fixed at 10^−12^, a value several orders of magnitude smaller than the temporal resolution of the analysis, and had no effect on the results.

The observation period spans from τ_x(min)_ to τ_x(max)_, corresponding to the lowest and highest TAT_X_ values among all n samples under observation, and the function ranges from 1 (when all sample TATs, t_i_^x^, are below τ_x(min)_) to 0 (when all sample TATs are higher than τ_x(max)_).

From a statistical standpoint, G_exp_(t) is equivalent to the empirical survival function of the TAT distribution. Indeed, the term inside the summation acts as a smoothed discrete indicator function, assuming a value close to 1 when t < τ_x_ and close to 0 when t > τ_x_. Consequently, G_exp_(t) represents the fraction of samples that have not yet been completed at time t, corresponding to the empirical survival function, S(t), i.e., the probability P that the TAT exceeds t. Since the survival function is complementary to the cumulative distribution function, this relationship can be expressed as S(t) = P(TAT > t) = 1 − F(t), where F(t) denotes the cumulative distribution function of the TAT values. Thus, G_exp_(t) can be interpreted as the empirical survival (or complementary cumulative distribution) function of the observed TAT distribution.

The DLS-inspired method is therefore not intended to introduce a new probability function, but to provide an operational discrete-time representation of sample clearance dynamics in automated laboratory systems. Within this framework, the principal novelty does not lie in the mathematical form of G_exp_(t), but in the definition of operational checkpoints that allow workflow performance to be monitored during the analytical process.

### 2.3. Model Fitting and Parameters Definition

Equation (1) resembles the correlation function used in DLS for cumulant analysis, originally proposed by Koppel in 1972, and derived from a series expansion of the exponential terms constituting the normalized molecular correlation function [30]. However, Equation (1) does not define a continuous function, as t is a discrete variable; therefore, the conventional canonical analysis used in DLS does not provide an adequate fit to the experimental data. Consequently, the data were fitted using the following Equation:

(2) $$G_{fit}\left(t\right)=\sum_{X=\alpha }^{\omega }\frac{\left|t-\tau_{X\left(min\right)}\right|-\left(t-\tau_{X\left(min\right)}\right)}{2\left|t-\tau_{X\left(min\right)}\right|+\varepsilon }\cdot \frac{n_{X\left(min\right)}}{n}+\frac{\left|t-\tau_{X\left(max\right)}\right|-\left(t-\tau_{X\left(max\right)}\right)}{2\left|t-\tau_{X\left(max\right)}\right|+\varepsilon }\cdot \frac{n_{X\left(max\right)}}{n}$$

where X ∈ {α, β, …, ω} denotes the sample classes (i.e., subgroups of samples sharing similar TAT values within the interval from τ_X(min)_ to τ_X(max)_). The ratios n_X(min)_/n and n_X(max)_/n represent the fractions of samples with TAT_X_ values closer to τ_X(min)_ and τ_X(max)_, respectively, with n = n_X(min)_ + n_X(max)_.

Equation (2) yields a broken line that does not reproduce the experimental points between τ_X(min)_ and τ_X(max)_, but correctly estimates the previously defined fractions of samples near the two limits (n_X(min)_ and n_X(max)_) exhibiting TAT_X(min)_ and TAT_X(max)_, respectively. Accordingly, the purpose of the fitting procedure is not to reconstruct the complete statistical shape of the TAT distribution, but rather to estimate the representative operational parameters associated with the predefined TAT limits.

The theoretical model was fitted to the experimental data by nonlinear optimization using the Microsoft Excel Solver tool. During the optimization, only the sample fractions n_X(min)_/n and n_X(max)_/n were iteratively adjusted to minimize the sum of squared differences (QSUMDIFF) between the experimental DLS curve described by Equation (1) and the theoretical model described by Equation (2) over the entire observation interval, whereas τ_X(min)_ and τ_X(max)_ were kept fixed as predefined operational constraints. The fitted curve is therefore not intended as the final output of the analysis, but as an intermediate mathematical tool for estimating the representative parameters.

The values of τ_X(max)_ and τ_X(min)_ are established before the fitting procedure and remain constant throughout the analysis. Specifically, τ_X(min)_ is experimentally determined as the minimum achievable TAT imposed by the analytical workflow, corresponding to the sum of the track transport time, analytical reaction time, and unavoidable technical processing time. Conversely, τ_X(max)_ is defined a priori by the laboratory as the maximum acceptable TAT according to the required clinical performance, beyond which a sample is considered to exhibit an abnormal processing delay. These values therefore represent fixed operational constraints of the analytical process and are not fitting parameters. Accordingly, n_X(min)_ and n_X(max)_ values are the only fitted parameters determining the proportion of samples displaying TAT_X_ comparable to the closest τ_X_.

The turning line at 50% (TL50, in minutes), defined as x = [τ_X(max)_ + τ_X(min)_]/2, corresponds to the median expected TAT_X_ between the two limits and, for symmetric distributions, to the expected mean TAT_X_. Accordingly, each entire subgroup X can be approximated as a bimodal distribution characterized by two representative TAT values, τ_X(min)_ and τ_X(max)_: the former includes samples with TAT_X_ ranging from τ_X(min)_ to TL50, while the latter includes samples with TAT_X_ ranging from TL50 to τ_X(max)_. TL50 therefore represents the expected time at which 50% of the samples will have completed their TAT. For multimodal batches, it corresponds to the expected TAT weighted by the number of samples belonging to each subgroup.

Since TL50 is defined from the predetermined values of τ_X(min)_ and τ_X(max)_, it represents a fixed operational checkpoint for a given analytical workflow. Consequently, the corresponding TLV50 parameter (defined in the following Section 2.4) should be interpreted and compared only under identical predefined operational conditions, that is, for the same τ_X(min)_ and τ_X(max)_ values. The purpose of the proposed framework is to assess changes in the experimental TAT distribution under fixed operational constraints rather than to evaluate the effect of varying these predefined limits.

Accordingly, the proposed framework is not limited to TL50 but can be readily extended to any user-defined operational checkpoint (e.g., TL25, TL75 or TL90), depending on the monitoring objectives of the laboratory. More generally, multiple different turning lines can be defined based on the expected TAT of critical samples. For example, to monitor slower sample TAT, a turning line can be drawn intersecting the *x*-axis at a value higher than TL50, such as TL75, defined by x = 0.75 [τ_X(max)_ + τ_X(min)_].

### 2.4. TLV50 Definition, Interpretation and Data Generation

The turning line value (TLV50) is defined as the *y*-coordinate of the intersection between the experimental curve G_exp_(t) and TL50, thus providing the fraction (or percentage) of samples (ranging from 0 (0%) to 1 (100%)) that are still under processing at TL50 time and, as a consequence, the fraction of samples that have not completed their TAT at this predefined checkpoint. For single-class samples with a symmetric TAT distribution, a value of 0.5 is expected. Values greater than 0.5 indicate that a larger fraction of samples has not yet completed their TAT at TL50 (i.e., delayed TAT), whereas values lower than 0.5 indicate faster TAT. Therefore, TLV50 reflects the relative delay of samples with TAT values exceeding TL50.

Unlike conventional summary metrics, such as the median TAT or the 90% completion time, TLV50 is conceived as an in-process operational indicator. Evaluated at the predefined checkpoint TL50, it quantifies the fraction of samples still under processing at a clinically meaningful stage of the analytical workflow, enabling deviations from the expected workflow to be identified before the target TAT is reached. To simulate the temporal evolution of TLV50 under buffering and non-buffering operating conditions, reflecting, in turn, system-dependent delay accumulation and management, TLV50 values (*n* = 250) were generated in Microsoft Excel using the built-in RAND() function, following the same procedure adopted for TAT data.

### 2.5. Multiclass Analysis

From a theoretical perspective, any number of distinct sample classes can be analyzed simultaneously, provided that their TAT distributions are sufficiently separated to allow independent estimation of the fitting parameters. This requirement is intrinsic to the fitting procedure, since substantially overlapping TAT distributions cannot be uniquely resolved into their individual class contributions. Consequently, simultaneous multiclass analysis is applicable only when the different classes are characterized by sufficiently distinct TAT ranges. In practice, under the same conditions, two or three sample classes (e.g., serum, heparinized plasma, EDTA plasma, citrate plasma, etc., comprising laboratory tests with similar TAT values) can typically be fitted simultaneously. When substantial overlap is present, independent correlation functions should be generated and analyzed separately for each class.

### 2.6. Application to Routine Complete Blood Count TAT Data

To demonstrate the applicability of the proposed methodology to real-world laboratory data, anonymized TAT data from 964 routine complete blood count (CBC) samples processed during a single working day from 8:00 A.M. to 5:00 P.M. were provided by the Clinical Chemistry and Pharmacology Laboratory at IRCCS Istituto Nazionale per le Malattie Infettive “L. Spallanzani” (Rome, Italy). The proposed methodology was applied to both the complete daily TAT dataset and the hourly TAT datasets, each comprising the samples entering the analytical workflow during a one-hour time interval. According to the predefined operational criteria described in Section 2.3, τ_X(min)_ and τ_X(max)_ were set to 1 min and 20 min, respectively, corresponding to the minimum time required to complete the CBC analytical process under normal operating conditions and the maximum acceptable TAT defined by the laboratory for the same analytical workflow. On this basis, TL50 was set to 10.5 min and used as the predefined operational checkpoint. TLV50 was calculated for both the complete daily dataset and each hourly dataset to quantify the fraction of samples still under processing at the TL50 checkpoint, thereby providing quantitative measures of both the overall daily workflow delay and its temporal evolution throughout the working day.

## 3. Results

### 3.1. DLS vs. Automated Laboratory Flow

A comparison of the principal characteristics (mathematical models approach and assumptions) of classical DLS and the parameters obtained from laboratory flow samples is reported in Table 1.

### 3.2. Correlation Functions and TAT Distributions

Different cases of TAT distributions (seven in total) are analyzed throughout the manuscript in the following Section 3.2.1, Section 3.2.2 and Section 3.2.3, progressing from the simplest case 1—a unimodal Gaussian distribution of TAT values—to more complex scenarios (cases 2–7), involving unimodal and bimodal random distributions with increasing levels of delay and sample-class complexity.

#### 3.2.1. Case 1—Unimodal Gaussian Distribution of TAT Values

**Figure 1:**
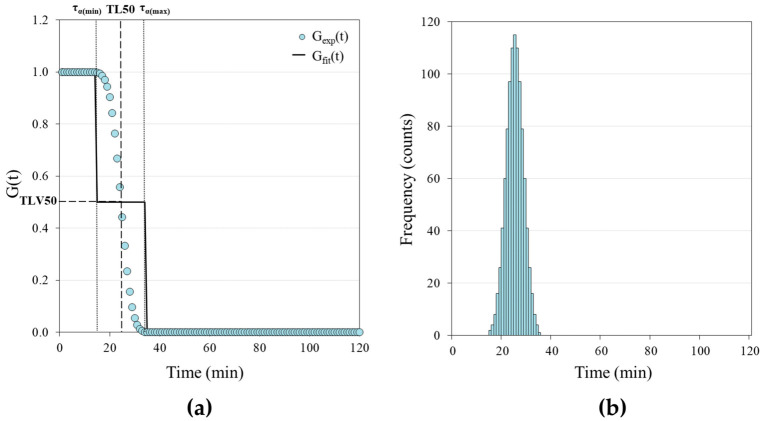
Representation of TAT values for a single class (X = α) under ideal unimodal Gaussian conditions (case 1, UG). (**a**) Experimental function G_exp_(t) (symbols, ○), generated according to Equation (1), and fitted function G_fit_(t) (solid line, —), obtained from Equation (2). τ_α(min)_, τ_α(max)_, TL50 and TLV50 are identified graphically as characteristic lines intersecting the curves (see main text for definitions and further details). τ_α(min)_ = 15 min, τ_α(max)_ = 35 min, TL50 = 25 min, TLV50 = 0.50. (**b**) Corresponding unimodal Gaussian distribution of TAT_α_ values for the same dataset. *n* = 1000.

Figure 1 illustrates the ideal unimodal Gaussian distribution of TAT values for a single class (X = α), hereafter referred to as the UG scenario.

In Figure 1a, the experimental function G_exp_(t), generated according to Equation (1), and the fitted function G_fit_(t), obtained from Equation (2), are shown together with τ_α(min)_, τ_α(max)_, TL50 and TLV50, while the associated Gaussian distribution of TAT is reported in Figure 1b. In Figure 1a, the fitted curve G_fit_(t) assumes that the parameter n_α_(t) is proportional to the number of samples with TAT_α_ = t. To reproduce the experimental curve using the bimodal form of Equation (2) (two populations of samples: A, with TAT = τ_α(min)_, and B, with TAT = τ_α(max)_), n_α_(t) is taken to be proportional to the number of samples with TAT_α_ values lower (n_α(min)_) or higher (n_α(max)_) than TL50. Accordingly, the n_α(min)_ samples display a TAT_α_ equal to τ_α(min)_ (population A), whereas the n_α(max)_ samples display a TAT_α_ equal to τ_α(max)_ (population B). The TL50, in the ideal unimodal Gaussian (i.e., symmetric) case, is expected to intersect G_exp_(t) at x = [τ_α(max)_ + τ_α(min)_]/2 and y = 0.5, indicating that exactly 50% of the samples have completed their process (TAT_α_ < TL50).

Finally, Table 2 provides a structured overview of the parameters of Equation (2) used to describe the models, classifying and defining them (as partly introduced in Section 2), and reporting their values for the simplest Case 1 (UG). Under perfectly symmetric conditions, n_α(min)_ and n_α(max)_ are both equal to 500, yielding TLV50 = 0.50. No samples exceed the τ_α(max)_ constraint, as indicated by the zero intercept between τ_α(max)_ and G_exp_(t). The n_α(min)_/n_α(max)_ ratio equal to 1.00 indicates a perfectly balanced distribution, with an equal number of samples having TAT_α_ values closer to τ_α(min)_ and τ_α(max)_, reflecting symmetric conditions with no shift toward shorter or longer times, and therefore no delays along the laboratory automation workflow.

#### 3.2.2. Cases 2 to 4—Random Unimodal Distributions of TAT Values

**Figure 2:**
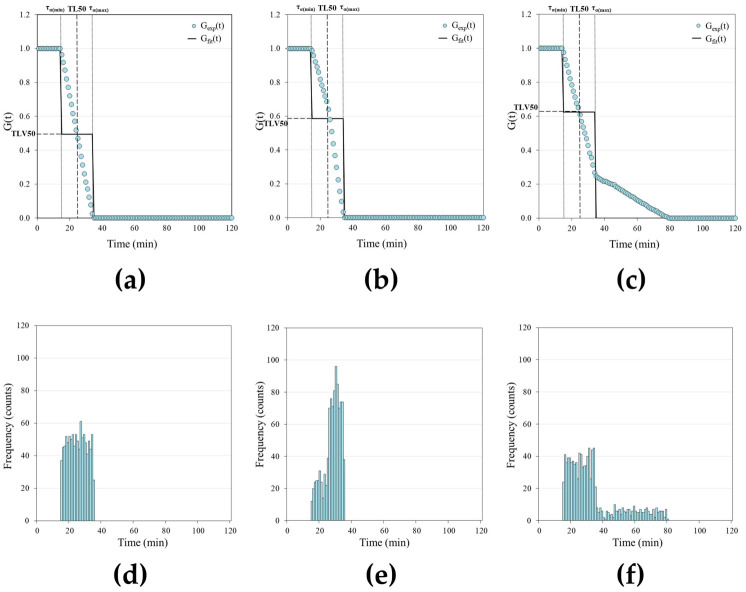
Representation of TAT values for a single class (X = α) under different unimodal random conditions, including scenarios without delays, with shifted TATs, and with delayed TATs. Panels (**a**,**d**), (**b**,**e**), and (**c**,**f**) correspond to Case 2 (UR), Case 3 (URs), and Case 4 (URd), respectively. (**a**–**c**) Experimental functions G_exp_(t) (symbols, ○), generated according to Equation (1), and fitted functions G_fit_(t) (solid line, —), obtained from Equation (2). τ_α(min)_, τ_α(max)_, TL50 and TLV50 are identified graphically as characteristic lines intersecting the curves (see main text for definitions and further details). For all panels: τ_α(min)_ = 15 min; τ_α(max)_ = 35 min; TL50 = 25 min; TLV50 = 0.49 (panel (**a**)), 0.59 (panel (**b**)) and 0.62 (panel (**c**)). (**d**–**f**) Corresponding unimodal random distributions of TATα values for the respective datasets. For each panel, *n* = 1000.

Figure 2 illustrates three different unimodal random distributions of TAT values for a single class (X = α), in contrast to the previously discussed ideal Gaussian case (UG, see Figure 1 and Table 2).

In Figure 2a–c, the experimental functions G_exp_(t), generated according to Equation (1), and the fitted functions G_fit_(t), obtained from Equation (2), are shown together with τ_α(min)_, τ_α(max)_, TL50 and TLV50, while the associated random distributions of TAT are reported in Figure 2d–f. Here, random data were generated to reproduce scenarios closer to real-world conditions; thus, while the Gaussian distribution in Figure 1 represents an idealized situation, these random scenarios more realistically reflect what may occur in an automated laboratory workflow in non-ideal conditions. Different scenarios are considered, highlighting how these distributions can exhibit delays of varying magnitude. Specifically, the following are represented:

- Case 2: unimodal random distribution of sample TAT values ranging from 15 to 35 min, without delays (shown in Figure 2a,d—UR scenario);
- Case 3: unimodal random distribution of sample TAT values ranging from 15 to 35 min, with about 50% of samples showing TAT values shifted toward longer times, while still remaining within the constraint range (shown in Figure 2b,e—URs scenario);
- Case 4: unimodal random distribution of sample TAT values ranging from 15 to 80 min, with about 25% of samples showing a delayed TAT higher than the τ_α(max)_ constraint (i.e., >35 min) (shown in Figure 2c,f—URd scenario).

Table 3 provides the parameters of Equation (2) for Cases 2–4 under unimodal random conditions. While the constraint parameters remain unchanged, the fitted values of n_α(min)_ and n_α(max)_ progressively deviate from the ideal symmetric condition of the UG scenario (*n* = 500). In Case 2 (UR), values remain close to symmetry (n_α(min)_ = 506, n_α(max)_ = 494; ratios 1.01 and 0.99), whereas in Case 3 (URs), a clear shift toward longer TAT values is observed (n_α(min)_ = 414, n_α(max)_ = 586; ratios 0.83 and 1.17). This trend becomes more pronounced in Case 4, where delayed samples dominate (n_α(min)_ = 376, n_α(max)_ = 624; ratios 0.75 and 1.25), with a high number of samples showing TAT values higher than the τ_α(max)_ constraint (i.e., >35 min). These changes, in terms of delay in the workflow, are reflected in the derived parameters: TLV50 increases from 0.49 (Case 2) to 0.59 (Case 3) and 0.62 (Case 4), while the fraction of samples exceeding τ_α(max)_, defined by the intersection between τ_α(max)_ and the G_exp_(t) function, remains 0% in Cases 2 and 3 and rises to 25% in Case 4. Consistently, the n_α(min)_/n_α(max)_ ratio decreases from 1.02 to 0.71 and 0.60, quantitatively describing the progressive transition from balanced to shifted and delayed distributions, thus reflecting increasing delays along the laboratory workflow chain.

#### 3.2.3. Cases 5 to 7—Random Bimodal Distributions of TAT Values

**Figure 3:**
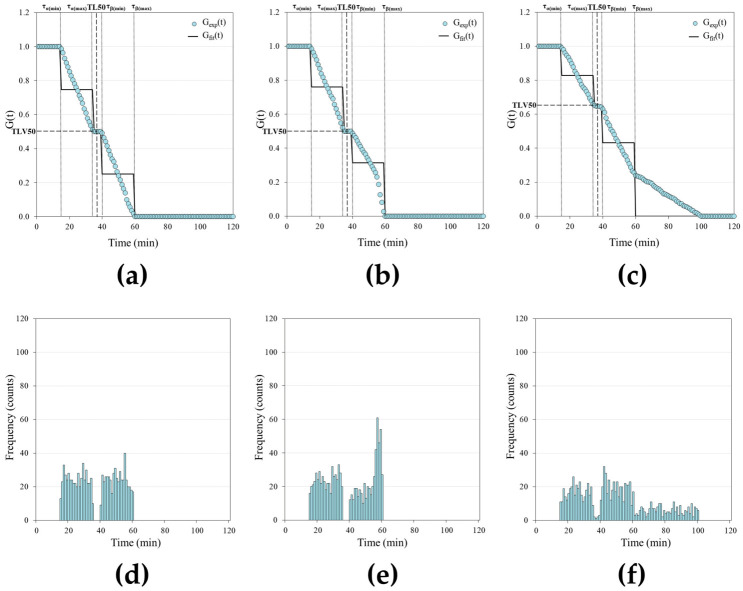
Representation of TAT values for two distinct classes (X = α, β) under different bimodal random conditions, including scenarios without delays, with shifted TATs, and with delayed TATs. Panels (**a**,**d**), (**b**,**e**), and (**c**,**f**) correspond to Case 5 (BR), Case 6 (BRs), and Case 7 (BRd), respectively. (**a**–**c**) Experimental functions G_exp_(t) (symbols, ○), generated according to Equation (1), and fitted functions G_fit_(t) (solid line, —), obtained from Equation (2). τ_α(min)_, τ_α(max)_, τ_β(min)_, τ_β(max)_, TL50 and TLV50 are identified graphically as characteristic lines intersecting the curves (see main text for definitions and further details). For all panels, τ_α(min)_ = 15 min; τ_α(max)_ = 35 min; τ_β(min)_ = 40 min; τ_β(max)_ = 60 min; TL50 = 37.5 min; TLV50 = 0.50 (panels (**a**,**b**)) and 0.65 (panel (**c**)). (**d**–**f**) Corresponding bimodal random distributions of TAT_α_ and TAT_β_ values for the respective datasets. For each (**d**–**f**) panel, *n* = 1000.

Figure 3 illustrates three different bimodal random distributions of TAT values for two distinct classes (X = α, β).

In Figure 3a–c, the experimental functions G_exp_(t), generated according to Equation (1), and the fitted functions G_fit_(t), obtained from Equation (2), are shown together with τ_α(min)_, τ_α(max)_, τ_β(min)_, τ_β(max)_, TL50 and TLV50, while the associated random TAT distributions are reported in Figure 3d–f. In these three cases, different profiles representative of non-ideal conditions in an automated laboratory workflow are analyzed:

- Case 5: bimodal random distribution of sample TAT values ranging from 15 to 35 min (X = α) and from 40 to 60 min (X = β), without delays (shown in Figure 3a,d—BR scenario);
- Case 6: bimodal random distribution of sample TAT values ranging from 15 to 35 min (X = α) and from 40 to 60 min (X = β), with about 20–25% of samples showing TAT_β_ values shifted toward longer times, although still below τ_β(max)_ (i.e., 60 min) (shown in Figure 3b,e—BRs scenario);
- Case 7: bimodal random distribution of sample TAT values ranging from 15 to 35 min (X = α) and from 40 to 60 min (X = β), with samples showing delayed TAT_α_ and TAT_β_ values, exceeding τ_α(max)_ (i.e., 35 min) and τ_β(max)_ (i.e., 60 min), respectively, up to 100 min (shown in Figure 3c,f—BRd scenario).

Table 4 provides the parameters of Equation (2) for Cases 5–7 under bimodal random conditions. For all models, the constraint parameters remain fixed (τ_α(min)_ = 15 min, τ_α(max)_ = 35 min; τ_β(min)_ = 40 min, τ_β(max)_ = 60 min; TL50 = 37.5 min; n_tot_ = 1000), while the fitted values progressively deviate from the ideal bimodal Gaussian condition (BG, 250 samples per subgroup). As indicated, in Case 5 (BR), the distribution is nearly symmetrical, with n_α(min)_/n_α(max)_ = 1.03 and n_β(min)_/n_β(max)_ = 1.00, and balanced between the two classes (n_tot(min)_/n_tot(max)_ = 1.01, with α and β each contributing 50% of the total population). TLV50 is equal to 0.50, with 50% of samples below τ_α(max)_ and no samples exceeding τ_β(max)_ (as indicated by the τ_α(max)_–G_exp_(t) and τ_β(max)_–G_exp_(t) intersections, equal to 0.50 and 0.00, respectively). In Case 6 (BRs), a shift toward longer TAT values is observed, particularly for the TAT_β_ population (n_β(max)_ = 314; ratio = 1.26), while the TAT_α_ class remains closer to symmetry (n_α(min)_/n_α(max)_ = 0.92). This results in an imbalance between early and late samples (n_tot(min)_/n_tot(max)_ = 0.74), although the overall class contribution remains equally distributed (50% α and 50% β). The invariance of TLV50 (0.50) between Cases 5 and 6 suggests a regime in which moderate shifts are absorbed without affecting the global balance of the system. Accordingly, 50% of samples remain below τ_α(max)_ and no samples exceed τ_β(max)_ (as evidenced by the τ_α(max)_–G_exp_(t) and τ_β(max)_–G_exp_(t) intersections, again equal to 0.50 and 0.00, respectively). In Case 7 (BRd), the asymmetry becomes more pronounced, with a clear dominance of delayed samples, especially in class β (n_β(max)_ = 433; ratio = 1.73). Population class α also shows reduced symmetry (n_α(min)_/n_α(max)_ = 0.94), while the internal imbalance is markedly stronger for class β than for class α. This is accompanied by a net redistribution of samples from class α to class β, with the total α population decreasing from 500 to 354 and β increasing to 647, and by a further imbalance between early and late samples (n_tot(min)_/n_tot(max)_ = 0.63). This is reflected in an increase of TLV50 to 0.65, marking a transition to a delay-dominated regime, with 65% of samples exceeding τ_α(max)_ and 24% exceeding τ_β(max)_, as reported for the τ_α(max)_–G_exp_(t) and τ_β(max)_–G_exp_(t) intersections, respectively). Notably, the increase in TLV50 is primarily driven by exceedance of τ_α(max)_, while only a fraction of samples surpass τ_β(max)_, indicating a progressive rather than abrupt propagation of delays along the workflow chain. Consequently, the relative contribution shifts toward class β (65%), while class α decreases to 35% of the total population.

### 3.3. TLV50 Temporal Evolution and Delay Progression in Simulated Buffering and Non-Buffering Systems

**Figure 4:**
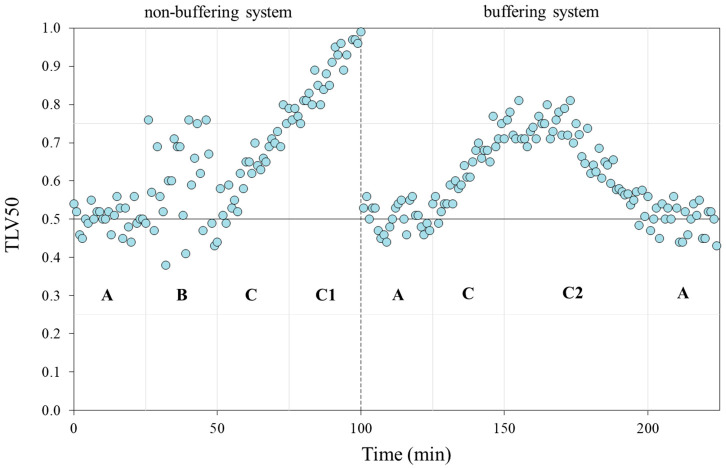
Time course of TLV50 under simulated non-buffering (**left**) and buffering (**right**) operating conditions. Labeled regions (A, B, C, C1, C2) identify representative operational states corresponding to uniform (A) and asymmetric (B) TAT distributions, as well as delay accumulation (C) with (C2) or without (C1) recovery and return to optimal workflow conditions. See main text for further details.

Figure 4 illustrates the time course of TLV50 values from simulated data (Section 2.4) under two distinct operating conditions (left: non-buffering system; right: buffering system), presented sequentially for comparison.

The labeled regions (A, B, C, C1, C2) represent different operational states:

- A: TLV50 fluctuations around 0.5 indicate a uniform distribution of sample TAT values within the τ_X(min)_–τ_X(max)_ range;
- B: deviations of TLV50 above or below 0.5 reflect an asymmetric TAT distribution, but the system can still recover to optimal conditions;
- C: a progressive increase in TLV50 indicates a reduction in sample processing rate and the onset of delay accumulation, leading to two possible outcomes (C1 and C2);
- C1: TLV50 increases continuously up to values approaching 1, reflecting persistent accumulation of delays due to unresolved issues in the TAT workflow (non-buffering system);
- C2: the attainment of a plateau followed by a decrease in TLV50, returning toward baseline values, indicates effective delay management, resolution of the issue, and restoration of stable and optimal operating conditions (buffering system).

Overall, the temporal evolution of TLV50 enables clear discrimination between persistent delay accumulation and adaptive system response. Similar considerations can be extended to systems involving multiple sample classes, where the relative proportions of different sample types influence the resulting correlation curves.

**Figure 5:**
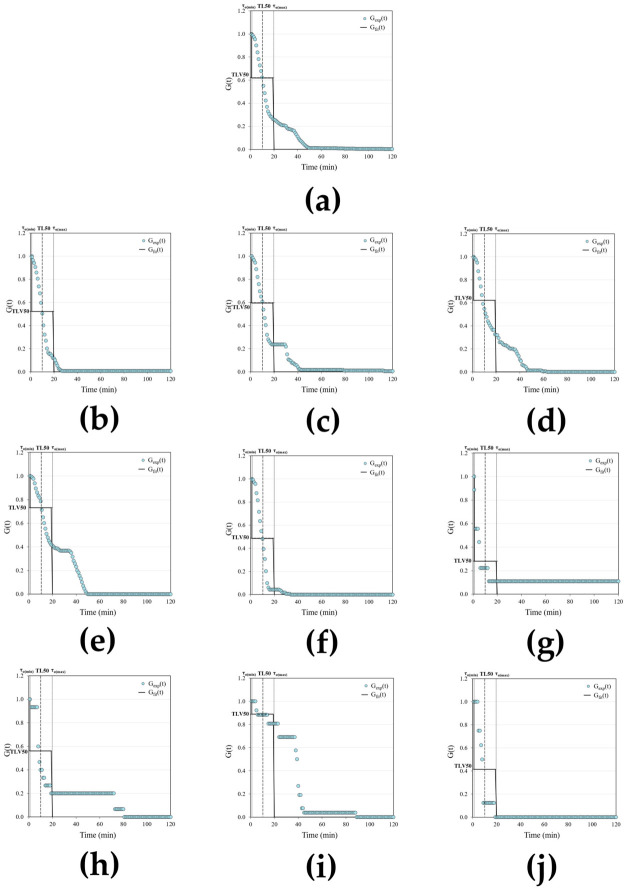
Application of the proposed methodology to routine CBC TAT data (X = α). (**a**) Analysis of the overall daily TAT dataset (from 964 CBC samples); (**b**–**j**) Analysis of the TAT values of CBC samples entering the analytical workflow during the following one-hour intervals: (**b**) 8:00–9:00 A.M.; (**c**) 9:00–10:00 A.M.; (**d**) 10:00–11:00 A.M.; (**e**) 11:00 A.M.–12:00 P.M.; (**f**) 12:00–1:00 P.M.; (**g**) 1:00–2:00 P.M.; (**h**) 2:00–3:00 P.M.; (**i**) 3:00–4:00 P.M.; (**j**) 4:00–5:00 P.M. In all panels, the experimental functions G_exp_(t) (symbols, ○), generated according to Equation (1), are shown together with the corresponding fitted functions G_fit_(t) (solid line, —), obtained from Equation (2). τ_α(min)_, τ_α(max)_, TL50 and TLV50 are identified graphically as characteristic lines intersecting the curves (see main text for definitions and further details). For all panels, τ_α(min)_ = 1 min; τ_α(max)_ = 20 min; TL50 = 10.5 min. TLV50 = 0.62 (panel (**a**)), 0.52 (panel (**b**)), 0.59 (panel (**c**)), 0.62 (panel (**d**)), 0.73 (panel (**e**)), 0.49 (panel (**f**)), 0.28 (panel (**g**)), 0.56 (panel (**h**)), 0.89 (panel (**i**)), 0.41 (panel (**j**)).

**Figure 6:**
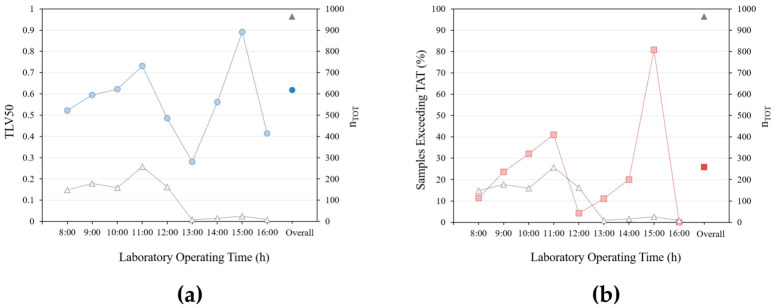
Hourly and overall daily workflow performance evaluated using the proposed methodology applied to routine CBC TAT data. (**a**) TLV50 values (blue ○) plotted as a function of laboratory operating time (in hours) with the corresponding number of CBC samples entering the analytical workflow during each hourly interval (n_TOT_) reported on the secondary *y*-axis (grey Δ); (**b**) Percentage of samples exceeding the predefined maximum acceptable TAT, τ_X(max)_ = 20 min (red □), plotted as a function of laboratory operating time (in hours) with the corresponding n_TOT_ values reported on the secondary *y*-axis (grey Δ). For simplicity, each hourly interval is represented by its starting time (e.g., 8:00 A.M. represents the 8:00–9:00 A.M. interval). In both panels, the darker symbols represent the corresponding overall daily values.

Accordingly, Figure 4 should be interpreted only as a conceptual illustration, whereas the practical applicability of the proposed framework is demonstrated using routine laboratory TAT data in Figure 5 and Figure 6.

### 3.4. Workflow Delay Assessment Using TLV50: Application to Routine Complete Blood Count TAT Data

As described in Section 2.6, TAT values from routine CBC samples (single class, X = α) were analyzed using the proposed approach.

Figure 5 shows the experimental functions G_exp_(t), generated according to Equation (1), and the fitted functions G_fit_(t), obtained from Equation (2), together with τ_α(min)_, τ_α(max)_, TL50 and TLV50. Figure 5a reports the analysis of the complete daily CBC workflow, including all of the 964 TAT values of the corresponding samples processed from 8:00 A.M. to 5:00 P.M. Moreover, Figure 5b–j show the corresponding hourly analyses, allowing visualization of the temporal evolution of the workflow performance throughout the working day. As expected, TLV50 varied among the hourly datasets, reflecting differences in the proportion of samples still under processing at the TL50 predefined operational checkpoint.

Figure 6 shows the hourly and overall daily time course of TLV50 values (blue ○) calculated using the proposed approach for the corresponding TAT of CBC samples entering the analytical workflow (n_TOT_, grey Δ) (Figure 6a), together with the corresponding percentage of samples exceeding the predefined maximum acceptable TAT (τ_X(max)_ = 20 min) (red □, Figure 6), calculated from the intersection between τ_X(max)_ e G_exp_(t). For simplicity, each hourly interval is represented by its starting time (e.g., 8:00 A.M. represents the 8:00–9:00 A.M. interval). The corresponding experimental and fitted correlation functions for each hourly dataset are shown in Figure 5b–j. Figure 6a shows that TLV50 progressively increased during the morning, reaching a first peak at approximately 11:00 A.M. (TLV50 = 0.73), indicating that 73% of the samples, instead of the expected 50%, were still under processing at the TL50 time (Figure 5e). This increase coincided with the period of highest routine CBC workload (n_TOT_ = 258), suggesting progressive accumulation of samples still under processing within the analytical workflow. However, as shown in Figure 6b, the corresponding percentage of samples exceeding predefined maximum acceptable TAT was 41%. At 12:00 P.M. (see also Figure 5f), the percentage of delayed samples decreased to 4%, indicating recovery of workflow performance despite the arrival of a new batch comprising 162 CBC samples. According to the classification proposed in Section 3.3, this behavior is consistent with a buffering system (Figure 4). At 3:00 P.M., despite the arrival of a small batch (n_TOT_ = 26), TLV50 reached a second, more pronounced peak (TLV50 = 0.89), indicating that 89% of the samples, instead of the expected 50%, were still under processing at the TL50 time; moreover, 80% of samples exceeded the predefined maximum acceptable TAT, τ_X(max)_ (Figure 5i). This peak coincided with the scheduled technician shift change. In Figure 6a, for the complete daily dataset (n_TOT_ = 964), the overall TLV50 was 0.62, compared with the expected value of 0.50, indicating that, on average, 12% of the daily workload remained under processing at the TL50 operational checkpoint (Figure 5a). Consistently, as illustrated in Figure 6b, the overall percentage of delayed samples was 26%, indicating that approximately one-quarter of the daily routine CBC workload exceeded the predefined maximum acceptable TAT (20 min).

## 4. Discussion

Dynamic light scattering (DLS) is widely used to determine particle size distribution in solution by analyzing temporal fluctuations of scattered light intensity, typically expressed through the photon auto-correlation function [25,26]. Once the autocorrelation function is obtained, different mathematical approaches can be applied to extract relevant information. The simplest approach assumes a single exponential decay. However, one of the most commonly used approaches is the cumulant method, which, in addition to the sum of exponentials described above, provides further information on the variance of the system [25,26].

In the present work, following the DLS theory, an autocorrelation function was generated for laboratory automated workflows and applied to specimens entering the analytical process to obtain clinically useful results, thereby describing their progression within the analytical phase of TAT. Specifically, a signal (value = 1) is assigned to each sample at increasing time channels until completion of the analytical process (i.e., results acquired, value = 0); the resulting signals are then summed and normalized to the total number of samples, yielding a correlation function that reflects the temporal evolution of sample processing. Conventional DLS methods are not suitable for describing this type of autocorrelation function. Therefore, a new mathematical model is proposed. Interpolation of the resulting curves provides quantitative parameters describing the relationship between observed and expected TAT. In particular, evaluation of TLV50 at the predefined checkpoint TL50 provides an operational indication of workflow status before the maximum admissible TAT (τ_X(max)_) is reached. If, at TL50, the fraction of samples still under processing is higher than expected (i.e., an abnormally high TLV50), this indicates that the analytical workflow is slowing down. Although these samples have not yet exceeded τ_X(max)_, the persistence of elevated TLV50 values suggests that, if the current trend continues, a proportion of samples is likely to exceed the predefined maximum admissible TAT. Thus, the predictive capability of the proposed methodology consists of providing an early operational warning of emerging workflow deterioration rather than prediction of the final TAT of individual samples. Unlike conventional TAT indicators, which are typically evaluated retrospectively after completion of the analytical process preventing timely corrective actions, the proposed framework enables workflow status to be assessed at predefined operational checkpoints (e.g., TL50) before the maximum admissible TAT (τ_X(max)_) is reached. This represents a novel approach for near real-time monitoring of laboratory efficiency, offering predictive insight into the ongoing sample analysis process and enabling timely identification of workflow deterioration.

Beyond providing quantitative workflow descriptors, the proposed model also offers considerable flexibility. It can be applied at different levels of the laboratory workflow, from individual steps to the entire process of clinical results production, including instruments, analytical procedures, track systems, or entire laboratory sections, enabling both localized and global performance assessment.

In this context, evaluation of TLV values, reflecting the temporal trend of sample delays, enables assessment of the system’s ability to manage and compensate for perturbations arising at any stage of the process. Based on this behavior, systems (instruments, laboratory section, entire lab, etc.) can be classified into two categories: buffering and non-buffering. Buffering systems are able to recognize and counteract disruptions, thereby limiting TAT delays through adaptive responses. These actions may involve different elements, including instrument control, informatics support, or operator intervention. In more complex systems, multiple elements may contribute to the overall buffering capacity. In contrast, non-buffering systems exhibit progressive delay accumulation due to the absence of effective compensatory mechanisms.

TLV-based analysis may enable comparison of different laboratory configurations, organizational models, and operational strategies, providing a quantitative framework for evaluating efficiency and performance. Parameters such as processing time, buffering capacity, and the proportion of delayed samples are inherently captured in the TLV temporal profile, supporting operational optimization and cost-effectiveness in healthcare systems.

The practical utility of the proposed methodology under real laboratory operating conditions was demonstrated through its application to routine CBC TAT data. The hourly TLV50 profiles successfully identified workflow perturbations associated with periods of increased analytical workload (e.g., morning peak workload) as well as organizational events (e.g., technician shift change), while the overall daily TLV50 provided a single quantitative descriptor of cumulative workflow performance. Because the same parameter can be calculated both for individual time intervals and for the complete daily dataset, the proposed framework enables objective comparison of laboratory performance within a working day as well as across different working days.

Building on these findings, the proposed approach could be implemented for real-time monitoring in highly automated laboratories by generating correlation curves at regular time intervals (e.g., every minute, hour, day, depending on the TAT scale of interest). For processes with TATs in the range of 10–40 min, for example, a new correlation curve may be generated every minute. In this framework, each batch of samples contributes to the correlation signal until all samples are processed (i.e., all TATs are generated), so that delayed samples prolong the contribution of the entire batch. As new samples enter and completed ones exit the system at each time interval, updated correlation curves are continuously generated. The temporal evolution of the associated parameters thus provides a real-time representation of system behavior, enabling continuous monitoring of workflow performance.

The flexibility of the proposed framework also opens the possibility for further methodological developments. One potential extension of the model is to consider each sample as a multi-component system, in which the completion of individual analytical tests contributes to a progressive, stepwise reduction of the overall signal (i.e., total TAT). In this framework, samples requiring different numbers and types of assays can be interpreted analogously to particles of different sizes in a polydisperse system. Consequently, the correlation signal may assume fractional values between 1 and 0, reflecting partial completion of the analytical process (e.g., a value of 0.5 when half of the required tests are completed). This extension would require more complex analytical functions but could provide detailed information on the contribution of individual tests, potentially enabling identification of specific assays responsible for TAT delays.

## 5. Conclusions

In conclusion, the proposed approach provides a practical operational framework for characterizing laboratory workflow behavior through the temporal evolution of sample processing. By enabling early identification of deviations from expected performance and characterizing the system response to workflow perturbations, it offers a new operational perspective for TAT analysis. The successful application of the proposed methodology to routine CBC TAT data demonstrates its feasibility under real laboratory operating conditions. If implemented within appropriate laboratory software, such as laboratory middleware or a laboratory information system (LIS), the proposed methodology could support continuous real-time workflow monitoring through automatic updating of the DLS-inspired function and its calculated parameters, enabling early identification of emerging workflow overload before the predefined maximum acceptable TAT is reached, thereby supporting timely operational intervention. Owing to its flexibility, the proposed framework can be applied at different levels of laboratory organization and may also serve as a quantitative tool for evaluating the impact of new analytical instruments, automation technologies, and organizational strategies on laboratory performance, resource utilization, and operational costs. Future studies will focus on validating the proposed methodology across different laboratories, analytical workflows, and automation platforms, as well as on systematic comparisons with established TAT monitoring methods using common large-scale datasets analyzed under identical operational conditions.

## Figures and Tables

**Table 1:** Conceptual and methodological comparison between dynamic light scattering (DLS) and the proposed laboratory automation approach for the analysis of sample turnaround time (TAT), highlighting differences in observed objects, time scales, mathematical treatment, extracted parameters, and underlying assumptions.

Parameter	DLS	Laboratory Automation
**Observed objects**	particles	sample TAT
**Time scale**	micro/nanoseconds	minutes
Number of objects required for statistical reliability *	10^3^–10^13^ particles depending on particle size	>30 laboratory samples
**Mathematical function used for data analysis**	cumulant analysis (sum of exponentials): ln(G_1_) = a + bt + ct^2^ + dt^3^ + et^4^ +…	sum of discontinuous functions (broken-line representation): G_fit_(t), see Equation (2)
**Extracted parameters**	mean particle size; particle size distribution (PSD); polydispersity index (PDI)	number of samples with TAT values closer to the expected minimum and maximum values; number of samples that have completed their process at a given time (TLV)
**Assumptions**	spherical particles and relatively narrow, monomodal sample distribution	no assumptions on TAT distribution shape; all simulations in this work were performed using values randomly distributed between τ_X(min)_ and τ_X(max)_

* The reported numbers refer to different statistical units. In DLS, statistical reliability is related to the number of scattering particles contributing to the autocorrelation function, whereas in the proposed methodology it refers to the number of laboratory samples included in the analysis. For laboratory automation applications, a minimum sample size of *n* > 30 was adopted as a conventional statistical reference for the theoretical simulations and does not represent a general requirement of the proposed methodology. Larger sample sizes may be required depending on the characteristics of the TAT distribution, particularly for the reliable characterization of skewed distributions and their tails.

**Table 2:** Parameters of Equation (2), classified as constraints, fitted, and derived quantities, with their definitions (see also Section 2.3 for further explanations) and corresponding values for the simplest case, namely the ideal unimodal Gaussian distribution (UG, X = α).

Parameter	Parameter Category	Definition (Unimodal Distribution, X = α)	Definition (Multimodal Distribution, X = α, β…)	Case 1 (UG)
**τ_X(min)_**	constraint	minimum expected TAT value for the sample class α [minutes]	minimum expected TAT value for each sample class X [minutes]	15 min
**τ_X(max)_**	constraint	maximum expected TAT value for the sample class α [minutes]	maximum expected TAT value for each sample class X [minutes]	35 min
**TL50**	constraint	expected time at which 50% of samples complete their TAT [minutes]	expected time at which 50% of samples complete their TAT, weighted by the number of samples in each class X	25 min
**n_tot_**	-	total number of samples entering the laboratory automation flow	total number of samples entering the laboratory automation flow	1000
**n_X(min)_**	fitted	number of samples with TAT_α_ = τ_α(min)_, including all samples with experimentally observed TAT_α_ values between τ_α(min)_ and TL50	n_tot(min)_ denotes the total number of samples (across all X classes) with TAT_X_ values lower than TL50	500
**n_X(max)_**	fitted	number of samples with TAT_α_ = τ_α(max)_, including all samples with experimentally observed TAT_α_ values greater than TL50	n_tot(max)_ denotes the total number of samples (across all X classes) with TAT_X_ values greater than TL50	500
**TLV50**	derived	fraction of samples (0.00–1.00) that have not completed their TAT_α_ at TL50	fraction of samples (0.00–1.00) that have not completed their TAT_X_ at TL50	0.50 *
**τ_X(max)_–G_exp_(t)** **intersection**	derived	intersection point between G_exp_(t) and τ_α(max)_ for sample class α: indicates the fraction of samples that have not completed their process within the maximum admissible TAT_α_	represents the fraction of all samples, irrespective of class, that have not completed their process within the maximum admissible TAT_X_ for the given class scenario (i.e., samples with significant delays)	0.00
**n_X(min)_/n_X(max)_**	derived	ratio of the total number of samples in class α with TAT_α_ values closer to τ_α(min)_ and τ_α(max)_	n_tot(min)_/n_tot(max)_ provides a general description of the multiclass laboratory workflow and the overall TAT distribution	1.00 **

* If TLV50 exceeds 0.50, this indicates delays in the laboratory automation workflow, although still within the maximum admissible TAT. Larger time shifts (>τ_X(max)_) are identified by the intersection between τ_X(max)_ and G_exp_(t). Alternative TLV thresholds can be defined according to different delay alert criteria. ** In the ideal case, the ratio approaches 1; lower values indicate a higher proportion of samples closer to τ_X(max)_ than to τ_X(min)_, reflecting delays in the automated flow.

**Table 3:** Parameters of Equation (2) for a single class (X = α) under unimodal random conditions (Cases 2, 3 and 4—UR, URs and URd scenarios, respectively). Constraint parameters (τ_α(min)_, τ_α(max)_, TL50) and total sample size (n_tot_) are fixed (which is the same as in the UG scenario), while fitted values of n_α(min)_ and n_α(max)_ are reported together with the ratios relative to the values from the UG scenario (500) and the associated ratios. Derived parameters (TLV50, τ_α(max)_–G_exp_(t) intersection, and n_α(min)_/n_α(max)_) are also included to quantify deviations from the ideal symmetric condition.

Parameter	Parameter Category	Case 2 (UR)	Case 3 (URs)	Case 4 (URd)
**τ_α(min)_**	constraint	15 min	15 min	15 min
**τ_α(max)_**	constraint	35 min	35 min	35 min
**TL50**	constraint	25 min	25 min	25 min
**n_tot_**	-	1000	1000	1000
**n_α(min)_** (ratio vs. UG) *	fitted	506 (1.01)	414 (0.83)	376 (0.75)
**n_α(max)_** (ratio vs. UG) *	fitted	494 (0.99)	586 (1.17)	624 (1.25)
**TLV50**	derived	0.49	0.59	0.62
**τ_α(max)_–G_exp_(t)** **intersection**	derived	0.00 (=0%)	0.00 (=0%)	0.25 (=25%)
**n_α(min)_/n_α(max)_**	derived	1.02	0.71	0.60

* For n_α(min)_ and n_α(max)_, the fitted values are compared to the ideal values of the UG scenario (i.e., the number of samples expected to belong to each class under perfectly symmetric conditions, *n* = 500); accordingly, the ratios (n_α(min)_/n_α(min)UG_ and n_α(max)_/n_α(max)UG_) are calculated with respect to these ideal values.

**Table 4:** Parameters of Equation (2) for two distinct classes (X = α, β) under bimodal random conditions (Cases 5, 6 and 7—BR, BRs and BRd scenarios, respectively). Constraint parameters (τ_α(min)_, τ_α(max)_, τ_β(min)_, τ_β(max)_, TL50) and total sample size (n_tot_) are fixed, while fitted values of n_α(min)_, n_α(max)_, n_β(min)_, n_β(max)_, n_tot(min)_ and n_tot(max)_ are reported together with the ratios relative to the values from the ideal bimodal Gaussian scenario (BG, *n* = 250). Derived parameters (TLV50, τ_α(max)_–G_exp_(t) and τ_β(max)_–G_exp_(t) intersections, n_α(min)_/n_α(max)_ and n_β(min)_/n_β(max)_, n_tot(min)_/n_tot(max)_, (n_α(min)_ + n_α(max)_)/n_tot_ and (n_β(min)_ + n_β(max)_)/n_tot_) are also included to quantify deviations from the ideal BG scenario.

Parameter	Parameter Category	Case 5 (BR)	Case 6 (BRs)	Case 7 (BRd)
**τ_α(min)_**	constraint	15 min	15 min	15 min
**τ_α(max)_**	constraint	35 min	35 min	35 min
**τ_β(min)_**	constraint	40 min	40 min	40 min
**τ_β(max)_**	constraint	60 min	60 min	60 min
**TL50**	constraint	37.5 min	37.5 min	37.5 min
**n_tot_**	-	1000	1000	1000
**n_α(min)_** (ratio vs. BG) *	fitted	254 (1.02)	240 (0.96)	172 (0.69)
**n_α(max)_** (ratio vs. BG) *	fitted	246 (0.98)	260 (1.04)	182 (0.73)
**n_β(min)_** (ratio vs. BG) *	fitted	250 (1.00)	186 (0.74)	214 (0.86)
**n_β(max)_** (ratio vs. BG) *	fitted	250 (1.00)	314 (1.26)	433 (1.73)
**n_tot(min)_**	fitted	503	426	386
**n_tot(max)_**	fitted	497	574	614
**TLV50**	derived	0.50	0.50	0.65
**τ_α(max)_–G_exp_(t)** **intersection**	derived	0.50 (=50%)	0.50 (=50%)	0.65 (=65%)
**τ_β(max)_–G_exp_(t)** **intersection**	derived	0.00 (=0%)	0.00 (=0%)	0.24 (=24%)
**n_α(min)_/n_α(max)_**	derived	1.03	0.92	0.94
**n_β(min)_/n_β(max)_**	derived	1.00	0.59	0.49
**n_tot(min)_/n_tot(max)_**	derived	1.01	0.74	0.63
**(n_α(min) _+ n_α(max)_)/n_tot_** **	derived	0.50	0.50	0.35
**(n_β(min) _+ n_β(max)_)/n_tot_** **	derived	0.50	0.50	0.65

* For n_α(min)_, n_α(max)_, n_β(min)_ and n_β(max)_, the fitted values are compared to the ideal values of the BG scenario (i.e., the number of samples expected to belong to each subgroup class under perfectly symmetric conditions, *n* = 250); accordingly, the ratios (n_α(min)_/n_α(min)BG_, n_α(max)_/n_α(max)BG_, n_β(min)_/n_β(min)BG_, and n_β(max)_/n_β(max)BG_) are calculated with respect to these ideal values. ** (n_α(min)_ + n_α(max)_)/n_tot_ and (n_β(min)_ + n_β(max)_)/n_tot_ quantify the fractional contribution of the α and β classes, respectively, to the total population.

## Data Availability

The raw data supporting the conclusions of this article will be made available by the authors on request.

## Abbreviations

The following abbreviations are used in this manuscript:

KPI	Key Performance Indicator
TAT	Turnaround Time
DLS	Dynamic Light Scattering
TL50	Turning Line 50
TLV50	Turning Line Value 50
UG	Unimodal Gaussian (Case 1)
UR	Unimodal Random (Case 2)
URs	Unimodal Random shifted (Case 3)
URd	Unimodal Random delayed (Case 4)
BG	Bimodal Gaussian
BR	Bimodal Random (Case 5)
BRs	Bimodal Random shifted (Case 6)
BRd	Bimodal Random delayed (Case 7)
CBC	Complete Blood Count

## References

[1] Hawker C.D. (2017). Nonanalytic Laboratory Automation: A Quarter Century of Progress. Clin. Chem..

[2] Genzen J.R., Burnham C.D., Felder R.A., Hawker C.D., Lippi G., Peck Palmer O.M. (2018). Challenges and Opportunities in Implementing Total Laboratory Automation. Clin. Chem..

[3] Al Naam Y.A., Elsafi S., Al Jahdali M.H., Al Shaman R.S., Al-Qurouni B.H., Al Zahrani E.M. (2022). The Impact of Total Automaton on the Clinical Laboratory Workforce: A Case Study. J. Healthc. Leadersh..

[4] Nam Y., Park H.-D. (2025). Revolutionizing Laboratory Practices: Pioneering Trends in Total Laboratory Automation. Ann. Lab. Med..

[5] Hawker C.D., Genzen J.R., Wittwer C.T., Rifai N., Horvath A.R., Wittwer C.T. (2017). Automation in the Clinical Laboratory. Tietz Textbook of Clinical Chemistry and Molecular Diagnostics.

[6] Yu H.-Y.E., Lanzoni H., Steffen T., Derr W., Cannon K., Contreras J., Olson J.E. (2019). Improving Laboratory Processes with Total Laboratory Automation. Lab. Med..

[7] Alhammad L.A., Ainosah T.K., Ahmad A.M., Samarkandi M.S., Jawi N.H., Alharthi M.A., Alsharif A.M., Anazi E.A.A., Aldugeshem S.A., Johali F.Y. (2023). The Impact of Laboratory Automation on Efficiency and Accuracy in Healthcare Settings. Int. J. Community Med. Public Health.

[8] Plebani M. (2025). Total laboratory automation: Fit for its intended purposes?. Clin. Chem. Lab. Med..

[9] Marzinke M.A., Clarke W., Marzinke M.A. (2020). Laboratory Automation. Contemporary Practice in Clinical Chemistry.

[10] Lou A.H., Elnenaei M.O., Sadek I., Thompson S., Crocker B.D., Nassar B. (2016). Evaluation of the impact of a total automation system in a large core laboratory on turnaround time. Clin. Biochem..

[11] Tsai E.R., Tintu A.N., Demirtas D., Boucherie R.J., de Jonge R., de Rijke Y.B. (2019). A critical review of laboratory performance indicators. Crit. Rev. Clin. Lab. Sci..

[12] Dawande P.P., Wankhade R.S., Akhtar F.I., Noman O. (2022). Turnaround Time: An Efficacy Measure for Medical Laboratories. Cureus.

[13] Vrijsen B.E.L., Haitjema S., Westerink J., Hulsbergen-Veelken C.A.R., van Solinge W.W., Ten Berg M.J. (2022). Shorter laboratory turnaround time is associated with shorter emergency department length of stay: A retrospective cohort study. BMC Emerg. Med..

[14] Pfaefflin A., Schuster K., Braun R. (2017). Short Centrifugation to Ameliorate Turn-Around-Time in Routine Coagulation Testing. Clin. Lab..

[15] Angeletti S., De Cesaris M., Hart J.G., Urbano M., Vitali M.A., Fragliasso F., Dicuonzo G. (2015). Laboratory Automation and Intra-Laboratory Turnaround Time: Experience at the University Hospital Campus Bio-Medico of Rome. J. Lab. Autom..

[16] Breil B., Fritz F., Thiemann V., Dugas M. (2011). Mapping Turnaround Times (TAT) to a Generic Timeline: A Systematic Review of TAT Definitions in Clinical Domains. BMC Med. Inform. Decis. Mak..

[17] Hawkins R.C. (2007). Laboratory Turnaround Time. Clin. Biochem. Rev..

[18] Steindel S.J., Howanitz P.J. (2001). Physician Satisfaction and Emergency Department Laboratory Test Turnaround Time: Observations Based on College of American Pathologists Q-Probes Studies. Arch. Pathol. Lab. Med..

[19] White B.A., Baron J.M., Dighe A.S., Camargo C.A., Brown D.F.M. (2015). Applying Lean Methodologies Reduces Emergency Department Laboratory Turnaround Times. Am. J. Emerg. Med..

[20] Pasqualetti S., Birindelli S., Aloisio E., Dolci A., Panteghini M. (2019). Clinical Governance Remains a Priority in Total Laboratory Automation Era. J. Appl. Lab. Med..

[21] Frater J.L., Anderson J. (2021). The impact of biosafety enhancement on stat laboratory quality metrics: Lessons from the COVID-19 pandemic. Clin. Chim. Acta.

[22] Vollmer R.T. (2006). Analysis of Turnaround Times in Pathology: An Approach Using Failure Time Analysis. Am. J. Clin. Pathol..

[23] Valenstein P. (1996). Laboratory Turnaround Time. Am. J. Clin. Pathol..

[24] Sung W. (2018). Brownian Motions. Statistical Physics for Biological Matter.

[25] Pecora R., Berne B.J. (2000). Dynamic Light Scattering: With Applications to Chemistry, Biology, and Physics.

[26] Schmitz K.S. (1990). An Introduction to Dynamic Light Scattering by Macromolecules.

[27] Arcovito G., Andreasi Bassi F., De Spirito M., Di Stasio E., Sabetta M. (1997). Dynamic light scattering study of fine semiflexible fibrin networks. Biophys. Chem..

[28] Di Stasio E., Romitelli F., Lancellotti S., Arcovito A., Giardina B., De Cristofaro R. (2009). Kinetic study of von Willebrand factor self-aggregation induced by ristocetin. Biophys. Chem..

[29] Di Stasio E., Bizzarri P., Bove M., Casato M., Giardina B., Fiorilli M., Galtieri A., Pucillo L.P. (2003). Analysis of the Dynamics of Cryoaggregation by Light-Scattering Spectrometry. Clin. Chem. Lab. Med..

[30] Koppel D.E. (1972). Analysis of Macromolecular Polydispersity in Intensity Correlation Spectroscopy: The Method of Cumulants. J. Chem. Phys..

